# Implementation of a self-management support approach (WISE) across a health system: a process evaluation explaining what did and did not work for organisations, clinicians and patients

**DOI:** 10.1186/s13012-014-0129-5

**Published:** 2014-10-21

**Authors:** Anne Kennedy, Anne Rogers, Carolyn Chew-Graham, Thomas Blakeman, Robert Bowen, Caroline Gardner, Victoria Lee, Rebecca Morris, Joanne Protheroe

**Affiliations:** NIHR CLAHRC Wessex, University of Southampton, Southampton, SO17 1BJ UK; Keele University, Staffordshire, ST5 5BG UK; University of Manchester, Manchester, M13 9PL UK

**Keywords:** Process evaluation, Normalisation Process Theory, Self-management support, Long-term conditions, Primary care

## Abstract

**Background:**

Implementation of long-term condition management interventions rests on the notion of whole systems re-design, where incorporating wider elements of health care systems are integral to embedding effective and integrated solutions. However, most self-management support (SMS) evaluations still focus on particular elements or outcomes of a sub-system. A randomised controlled trial of a SMS intervention (WISE—Whole System Informing Self-management Engagement) implemented in primary care showed no effect on patient-level outcomes. This paper reports on a parallel process evaluation to ascertain influences affecting WISE implementation at patient, clinical and organisational levels. Normalisation Process Theory (NPT) provided a sensitising background and analytical framework.

**Methods:**

A multi-method approach using surveys and interviews with organisational stakeholders, practice staff and trial participants about impact of training and use of tools developed for WISE. Analysis was sensitised by NPT (coherence, cognitive participation, collective action and reflective monitoring). The aim was to identify what worked and what did not work for who and in what context.

**Results:**

Interviews with organisation stakeholders emphasised top-down initiation of WISE by managers who supported innovation in self-management. Staff from 31 practices indicated engagement with training but patchy adoption of WISE tools; SMS was neither prioritised by practices nor fitted with a biomedically focussed ethos, so little effort was invested in WISE techniques. Interviews with 24 patients indicated no awareness of any changes following the training of practice staff; furthermore, they did not view primary care as an appropriate place for SMS.

**Conclusion:**

The results contribute to understanding why SMS is not routinely adopted and implemented in primary care. WISE was not embedded because of the perceived lack of relevance and fit to the ethos and existing work. Enacting SMS within primary care practice was not viewed as a legitimate activity or a professional priority. There was failure to, in principle, engage with and identify patients' support needs. Policy presumptions concerning SMS appear to be misplaced. Implementation of SMS within the health service does not currently account for patient circumstances. Primary care priorities and support for SMS could be enhanced if they link to patients' broader systems of implementation networks and resources.

**Electronic supplementary material:**

The online version of this article (doi:10.1186/s13012-014-0129-5) contains supplementary material, which is available to authorized users.

## Introduction

Implementing self-management approaches have the potential to improve health outcomes and reduce the fiscal burden on health care systems [1]. Current adoption of self-management support (SMS) has been mainly directed at patient self-skills training and behaviour change with little consideration of the concurrent activities required of multiple partners in whole health system implementation to ensure adoption and integration into long-term condition management [2]. The disconnection of patient education skills training from chronic disease management located in primary care settings is likely to have contributed to a lack of reach to those most likely to benefit [3],[4]. Whilst primary care has been identified as a key provider of self-management education and support because of its reach and increasingly central role in chronic disease management [5],[6], general practitioners (GPs) have been reluctant to refer to external self-management education programmes because of a fear of fragmenting care and ambiguity over patient benefit [7]. Targeting and personalised management along with patient-mediated strategies are advocated components of future SMS interventions [8],[9].

Long-term condition management currently operates in UK primary care through an increasingly biomedical, bureaucratic, specialised and reductionist framework [10], reinforced by the Quality and Outcomes Framework (QOF) [11] and pay-for-performance schemes [12]. A counter trend is the advocation of empowering and engaging patients in their own care, a more patient-centred, social and psychological model of care. However, an implementation gap has been identified between these national policy aspirations and current means of delivery as patients are not being directed to local resources or engaged in behaviour change [13],[14]. The components of the Whole System Informing Self-management Engagement (WISE) approach to SMS had been firmly established but not implemented in a primary care context. Thus, there is a need to understand how a systemic patient-centred approach to SMS reconfigures existing relationships, communication and practices and how (and if) the principles of a whole systems approach can translate, embed and integrate into routine practice [15],[16]. The implementation literature states the importance of being clear about specifying the implementation strategy used [17]. The whole systems education strategy is outlined in Table 1.

**Table 1:** **Specification of WISE implementation strategy**

	Specification of the implementation strategy
Actors	The organisation: employs and supports lay trainers to deliver WISE to whole practice teams
	Health care professionals: once trained, use WISE approach with their patients
Actions	Practice teams given knowledge, skills and tools to improve self-management support
Action targets	The organisation: facilitates training process (funding for training and employment of trainers), access to community resources (online directory of self-care organisations), develops management strategy, finds local GP champion
	The practice: commit whole practice to attending training, nominate two practice champions for WISE, develop systems to ensure tools accessible to staff and patients, work with trainers in follow-up sessions to embed WISE, share and discuss learning within practice teams
	Practice staff: use WISE approach knowledge, skills and tools to provide tailored support for self-management
	Patients: given PRISMS form and informed of a change of approach by practice staff to help them manage their condition
Temporality	Assumption that practice staff would start to use WISE approach with patients with long-term conditions after completing training
Dose	Two training sessions of 3 h 1 month apart. Intermediate session and post-training support with trainers offered
	Session 1: 3 h whole practice—GPs, nurses and administrative staff
	Brief introduction to WISE
	Care pathways exercise—mapping the process of care from reception to self-management
	Interactive session—making the WISE tools work in your practice:
	PRISMS form (Patient Report Informing Self-Management Support): designed to encourage patients to reflect on their support needs, how they were managing and which symptoms and illness-related matters required attention in their everyday lives. Patients' priorities to form a basis for negotiated decision-making and tailoring access to appropriate information or resources
	Guidebooks developed with patients to provide experientially based information, alongside medical evidence about treatment options [18],[19]. The guidebooks were intended to encourage patients to consider changes they could make to manage their condition
	Online directory of local services developed by the PCT providing up-to-date information about community services, support groups and education programmes. Linking to:
	Group training and support (Expert Patients Programme courses, group education, exercise classes)
	Voluntary sector and local support (patient support groups, health trainers)
	Session 2: 3 h clinicians—GPs and nurses
	Refresh on WISE approach
	Show DVD giving examples of WISE approach consultations plus discussion
	Skills training—role play to practice three core skills:
	How to assess what each patient can do and needs to do
	How to share decisions with patients
	How to make sure patients get the right support
	Discussion on how to ensure sustainability of WISE
Implementation outcome affected	Adoption and feasibility of the WISE approach at organisation, practice, professional and patient level
Justification	Using NPT to explain how new or modified practices of thinking, enacting and organising work associated with WISE are operationalised in health care

Education strategy.

Training of mixed practice team (GPs, nurses and administrative staff) in using a structured whole systems approach to target and improve within practice communication, professional-patient communication, patient education and advice and patient self-management outcomes.

**Figure 1:**
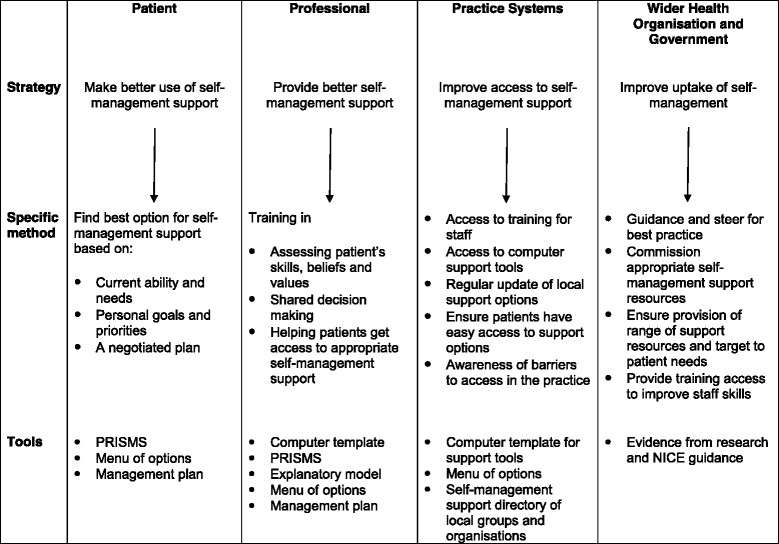
**Model for WISE approach.**

The WISE approach [20] is an intervention with evidence-based components, designed to provide and encourage SMS uptake and delivery across a whole health system [21]. Informed by an understanding of how health care professionals and patients respond to long-term conditions, WISE aspired to engage patients, practitioners and the service organisation using a structured approach (Table 1) [22]. For this paper, we also considered the outer systems which influence implementation [23] and have added to the original model (see Figure 1) [22]. A large-scale randomised controlled trial (RCT) designed to test effectiveness and cost-effectiveness found that WISE had no effect whatsoever on 12-month patient outcomes [22],[24],[25]. The process evaluation explains why this evidence-based approach was not implementable in routine primary care.

The WISE intervention focussed on training primary care teams (GPs, nurses and administrative staff) over two half-day sessions and providing tools to support self-management. The exemplar conditions for the trial were diabetes, chronic obstructive pulmonary disease (COPD) and irritable bowel syndrome (IBS). A primary care trust (PCT)^a^ was the organisation supporting and investing in its implementation. The PCT employed two experienced lay trainers to deliver and support the training and practices. The PCT is in the North-West of England and serves a socio-economically deprived population. The training is described in detail elsewhere [25]. The tools included PRISMS [26], Guidebooks [18],[19] and an online directory (see Table 1).

Development and evaluation of the intervention followed the MRC framework for complex interventions. The pre-clinical and development phase was informed by theory and evidence set in the context of policy expectations and guidance. There was good evidence of effectiveness for the components of the intervention (patient-centred information, shared decision-making and training of health professional teams in secondary care) [26]-[39]. A formative evaluation was used to evaluate the feasibility of the intervention in a primary care setting and refine training prior to roll-out in an RCT [25]. The process evaluation was pre-specified to complement and provide additional evidence to the main trial [22],[40].

### Process evaluation question

What are the barriers and facilitators which affect the implementation of WISE at patient, clinical and organisational levels?

The conceptual framework for development and evaluation was twofold. Firstly, WISE was developed from mixed methods research which investigated the circumstances and components where patient-centred self-management was likely to be most effective [26]-[39]. Secondly, Normalisation Process Theory (NPT) [41] had utility for sensitising the research to the reaction, incorporation or rejection of WISE from a service user and professional perspective.

NPT as a theory of implementation is orientated to explain how new or modified practices of thinking, enacting, and organizing work associated with WISE are operationalized in healthcare. The theory is concerned with three core problems.

1. Implementation—the social organization of bringing practices into action.
2. Embedding—the processes through which practices do or fail to become routinely incorporated in everyday work of individuals and groups.
3. Integration—the processes by which practices are reproduced and sustained among the social matrices of an organization.

Practices become routinely embedded—or normalized as the result of people working, individually and collectively, to enact them. This is promoted or inhibited through the following generative mechanisms through which human agency is expressed:

- *'Coherence'* refers to the extent that a technology or health practice must make sense to targeted stakeholders.
- '*Cognitive participation'* concerns the commitment and collective engagement of stakeholders.
- *'Collective action'* refers to the relationships and work required enabling a new intervention to be taken up in practice and identifying the barriers to implementation and embedding.
- '*Reflexive monitoring'* holds that successful embedding of resources and technologies in everyday practice relies upon a continuous process of evaluation to feedback and refine the object of implementation.

A key objective is 'How is this new initiative translated and implemented in practice?' which refers to two key issues: the implementation of training in the WISE approach and the implementation of tools to assess patient priorities (e.g. PRISMS forms).

Where results are positive, evidence is required to identify 'active ingredients' aiding generalisability and facilitating learning and translation into everyday practice. Where results are negative or inconclusive, evidence is needed to identify sources of failure and stasis. In other words, why did promising theory not translate into practice? It is important to identify what works well for which practices, individuals and stakeholders and in what context.

### Aims of the process evaluation

- To explore organisations', professionals' and patients' attitudes and responses to the costs and benefits of implementing WISE
- To explore patient perspectives about and engagement with existing service management arrangements and the nature of interaction with professionals
- To explore patient attitudes to engagement with new self-management arrangements
- To examine changes in personal management arrangements, impact on existing caring relationships and use of additional services and resources

## Methods

We viewed each level of implementation as part of a multilevel case study with an overarching analysis. NPT formed the bases of the process evaluation methodology and analysis; the survey questionnaire and the interview schedules were orientated around NPT constructs to gain a view on how WISE was being operationalised and actioned across settings. Responses to the WISE approach were determined at three levels (see the Additional file 1):

1. Organisational level (sub-divided into the health organisation and the general practice)
  Acceptability to the Health Organisation. Baseline face-to-face interviews with a purposive sample representing PCT governance bodies and those key to the roll-out of WISE were digitally recorded.
  Acceptability to practices and recruitment to the trial. Assessment methods included contemporaneous trainer and researcher notes, e-mails from practices and minutes from meetings.
2. Practice staff level (primary care)
  Experience of the training-post-training evaluation questionnaire collected immediately after each session.
  Questionnaire to survey use of tools and enrolment in the WISE approach conducted 6 months post-training and posted out to practices with accompanying pre-paid return envelopes.
3. Face-to-face in-depth interviews with practice staff in trained practices. Three to six months following training, all staff were invited to take part in semi-structured interviews, and data collection continued until a broad representation of practice types (based on practice size, population served and number of GPs) was reached. Practice staff interviewed included GPs, nurses, the practice manager and the administrative staff. The interviews were digitally recorded.
4. Patient level. In-depth interviews with a purposefully selected sample of trial participants. Twenty-four patients were selected using a maximum variation sampling strategy based on the trial baseline data: condition, length of time diagnosed, number of GP contacts, self-efficacy scores, help and support from family, choices ever offered by GP (based on the question 'I feel that my doctor has provided me with choices and options'), age and gender.

Interviews lasted between 40 to 90 min.

### Analysis

The interviews were transcribed and then read and coded by members of the research team (based on the questions in Table 2) who provided written comments and interpretations of exemplar quotes. In line with how others have used NPT to provide a conceptual framework [42]-[44], coding reliability was established through a series of team meetings. This emergent data was collated to create a wall-chart to map NPT constructs. The adequacy of the mapping and data interpretation were established through discussion with all co-authors and refined in a data analysis workshop with an external expert (Carl May). The chart was finessed to produce the final version of Table 2. A team-based iterative process drawing on the quantitative data allowed an understanding of the implementation at each level. Discussions within the team then contributed to the final overarching analysis.

**Table 2:** **NPT framework**

NPT construct	Component	Questions to consider	Organisation	Professional	Patient
Coherence: sense-making work	Differentiation	Does the stakeholder (SH) recognise the WISE approach as different from their existing ways of working?	New type of grant for PCT—needed new skills/management/finance to embed	Difficulty differentiating WISE principles from those underpinning existing practice undermined the embedding of the intervention. SMS/WISE not seen as different to their perception of how they already work	Does not see benefit in getting SMS from health practitioners
		Does the SH understand the purpose of self-management support (SMS)?	SMS fits with the direction the PCT wants to move in		
	Communal specification	Does the SH recognise the steps s/he needs to take to assist in the integration of WISE?	Top/down initiative—needs to be embedded in 'right' part of the organisation	Limited communication within practices post-training stifled discussion surrounding WISE and its potential benefits	Not prepared to initiate SMS discussion with GP/nurse
			Middle management not involved		
	Individual specification	Does the SH identify their personal role and responsibilities with the WISE approach?	Pretty clear roles for people—lack of ownership by middle managers	Marked variation existed in nurses' opinion as to the fit of the WISE tools in their current practice: the guidebooks fitted well and PRISMS did not	SM responsibility seen as outwith interactions with health service
	Internalisation	Does the SH identify any benefit in adopting the WISE approach and for whom?	Recognition of PCT as innovative org—approach seen as beneficial to population	Familiarity with information and services provided by long-established, reputable sources undermined effort applied to identify the benefits and value of the WISE guidebooks. One nurse saw WISE as improving patient care and relationships	Guidebook useful—to compare with others
Cognitive participation: relational work	Initiation	To what extent does the SH appear to have been a supporter of the process to integrate WISE?	Champion SMS innovations for some time—WISE fits this—self-care team and EPP and tele care	Failure to engage in a practice-wide strategy discouraged individual commitment to adopt WISE. QOF is priority	Does not see point of engaging with HCP about SMS
	Enrolment	Has the SH made any adaptations to their personal routine or assisted in the reorganisation process leading to implementation?	Paid for dedicated trainers—supported practices to attend training	In most cases, no adaptations were made, but nurses who saw themselves as having autonomy were able to take up the WISE tools in individual practice	None—did not take PRISMS forms to GP
	Legitimation	Does the SH believe that it is appropriate for them to be involved in integrating WISE?	Yes—a key aim for the PCT but doubts from some over cost benefit ratio. Evidence base not legitimate, not relevant to GPs—new elements Step up	Many nurses did not perceive their roles required adoption of the WISE approach.	No
	Activation	Has the SH taken steps to sustain the use of WISE?	Implemented training within a self-care team in hopes of sustaining	Assessment and review of the processes involving the tools to sustain their use was afforded little priority, too many reasons not to use PRISMS and QOF the over-riding practice priority	No
Collective action: operational work	Interactional workability	What work does the SH describe as having taken place to operationalise the use of the WISE approach?	In terms of grant—needed to work on getting budget right. Managed through professional directorate NOT commissioning	Difficulty engaging patients in self-management practices limited enthusiasm to invest effort in new ways of working. PRISMS used (rarely) to open up consultation, but not to take the next step of supporting behaviour change	None, concerns around disrupting the status quo of relationships
			Trainers and SC team		
			Creation of online directory		
	Relational integration	To what extent does the integration of the WISE tools and resources help or impede people's work?	Needed management champion to ensure correct pathways—did not happen	The convenience and ready access to information in hard-copy format encouraged use of the guidebooks but PRISMS got in the way of existing tasks and priorities	Guidebook helped to consider SM choices in day to day life outwith HCP
					PRISMS might be a prompt sheet
	Skill-set workability	Who does the SH view as being best placed to make use of the WISE approach?	PCT had to get new skills in managing research budget. Trainers to support and spread the word; training skills facilitative and reflexive	Nurses delegated SMS by the GPs. But this work is hidden and not audited. Responsibilities as health educators promoted nurses' role as implementers of WISE's holistic approach to SMS. Books most compatible and accentuated patient-centred approach	SM skills still seen as individual responsibility and trial and error – hard to see where HCP fits in
		How compatible is the WISE approach with their current tasks?	Needed to be in commissioning directorate to work		
	Contextual integration	Does the integration of WISE fit with the objectives of the organisation/individual?	Yes—innovative PCT at forefront of policies directed at deprived population	QOF is the priority of the practice and nurses happy to do the tasks but the tensions are with the skills they see themselves as having which are disregarded by the QOF process. QOF tick-box priority means no space for SMS work. The practice systems were not able to integrate PRISMS forms—so 'not to hand'	No
Reflexive monitoring: appraisal work	Systematisation	Has the SH taken practical steps to measure the influence of adopting the new techniques?	No and at a loss as to how to do this, see it as pilot. No outcomes to measure, not audited, GPs not accountable	Limited, informal gathering of feedback from patients regarding the accessibility and utility of the WISE guidebooks was recorded, suggesting that some use this resource as a prompt and practical means of disposal when responding to patient concerns	No
	Communal appraisal	Are there any joint efforts to appraise the impact of implementation?	Costly model (training individual practice)—seen as not viable	No—reflecting a silo-style working environment, few practitioners recorded engaging colleagues in discussion of their experience of using the tools	No
	Individual Appraisal	Does the SH reflect personally on the impact of the WISE approach on his/her routine?	Trainers kept reflexive journal and communicated with research team	The limited take up of the tools and resources was reflected in the prevalent view that the training had produced little change in practice. In contrast, supporters of PRISMS noted the positive impact on patient engagement	No as no impact
	Reconfiguration	Has the SH made attempts to modify the way the WISE approach is used as a result of experience?	Trainers worked with research team to adjust training content	For adopters of PRISMS, identifying how the process of using it could be adapted to fit in with existing practice such as by focussing on the most pressing concern rather than a range of issues was important to the sustainability of the tool	No

### Ethical considerations

The study was approved by the Salford & Trafford Local Research Ethics Committee, REC reference number 09/H1004/6.

## Results

Table 2 summarises the findings across the three levels using NPT constructs to illuminate for whom which elements of WISE did or did not work in practice. This analysis explains why there were no effective changes in personal management arrangements, existing care "as usual" relationships or use of additional services and resources.

### Health economy system level readiness for embedding SMS

Seven key individuals were interviewed at the start of the roll-out of WISE. These included three PCT senior managers (including the chief executive), the NIHR programme grant PI and project manager and the two WISE trainers.

#### Whose idea was this?

The PCT was selected for WISE implementation because of previous support for self-management initiatives and its research-friendly identity. At the time of the study, the PCT operated as a health economy with close geographical and local networks; managers were highly motivated and ambitious, with a strong local identity and not afraid of innovation, key characteristics in facilitating senior management's buy-in to the WISE project.

A piece of work like this is fundamentally important to us. It's pretty good commonsense really isn't it that, you can achieve the win/win of getting the best economic impact of your investments but also getting the best impact in terms of quality of services provided to patients and to citizens to get it right on the preventative aspect of the agenda rather than having to deal constantly with exacerbations and funding expensive health care interventions. (Senior manager)

There were reports of tensions arising from the way involvement in the project had been disseminated from executive level to middle manager level and about ownership and credit. PCT managers at executive level were committed,^b^ but WISE was not established in the Commissioning Directorate (the point in the NHS system where planning, agreeing and monitoring of services occurs) making it difficult to get buy-in from the commissioning managers and for WISE to be integrated with the PCT's annual planning cycle.

In terms of ownership and it's been difficult because once you've got a business plan for the year for your own department and you've got your resources highlighted for where you're going to put your energies that year … It's then very difficult to pull that team of people off that programme work onto something that they've had no involvement or engagement with at the outset and the planning stages. (Senior manager)

The lack of an appropriate 'home' for WISE or a champion working at managerial level had consequences for its profile across the health economy. The status of WISE as a research project (a pilot rather than mainstream activity) meant that managers were uncertain as to its future which resulted in ambivalence to engage with it. The imminent changes around commissioning exacerbated these uncertainties.

WISE was eventually allocated to the long-term conditions commissioning manager. However, this manager did not seem to demonstrate 'ownership' of WISE.

At the moment, I'm just taking a high level view of it, …because it's being steered really and managed through [senior manager], rather than through commissioning. (Senior manager)

The lack of ownership by managers was a challenge for the trainers, who—whilst employed by the PCT—had no managerial support or interest shown in their work.

It has felt to me as if WISE has slipped the radar of somebody…….it's nobody's baby in the PCT. (Trainer)

#### Breaking the norms of training

The training model was directed at a whole practice using a learning organisation ethos. This differed from traditional professional education [45],[46]. Training was not didactic but facilitative, flexible and encouraging of reflection; for some, this made the WISE approach less likely to be built into practice.

I think the WISE training is a totally different type of training. I don't think it's like any other training you have, so most of the training we have is very factual, it's, "Don't use that for diabetes any more, that's old hat; this is what we want you to do now and these are the new targets." …whereas I suppose your WISE training is a whole different concept, really. I still think there's room for being talked at a little bit, … But I don't think you will change people in just two sessions. (GP practice 22)

Follow-up sessions were intended to ascertain whether the training translated into changed clinical behaviours but trainers had difficulty in getting access to practices.

It was always oh we haven't done anything yet. And we had numerous meetings actually cancelled, so it'd be oh sorry, the doctor's busy, can you make it next week' So there isn't really an interest. (Trainer)

### Practice readiness for embedding

#### Not our priority

Reactions to training ranged from interest and enthusiasm to disinterest and apathy. WISE was not viewed as core or fitting with pay-for-performance targets which did not include delivering SMS. A perceived need for training in SMS was lacking, so the approach to engaging practices stressed that the PCT would meet training costs (funding for locum and out-of-hours cover) and targeting research-friendly practices early on to encourage more reluctant GPs to join the programme. It was often the practice manager who drove the decision to be involved rather than the GPs.

I would have thought it was more [practice manager], if she thinks it's beneficial to the practice she would be on board, if she feels that other surgeries are participating and it helps them then she would be on board. (GP practice 2)

Self-management was not a priority for practices, and there was a lack of conviction that SMS would be effective. Significantly, the WISE approach was not seen as providing anything novel. Staff claimed that they were already providing good care for patients with systems and strategies for long-term conditions in place as evidenced by performance in QOF, so, changing practice offered no tangible benefits to them. This response was seemingly linked to a limited view about what constitutes adequate provision of chronic disease management in primary care which appears to be rationed and focussed on biomedical markers. Additionally, patients were viewed as unlikely to take up or benefit from a self-management approach. References to the responsibility patients take for their care act as a marker about the acceptable division of labour between professionals and patients.

I try and do the self-care management where I possibly can but I only have 15 minutes and in that could have been asthma checks, it could be a BP check bloods, height, weight, BMI, depression screening, geriatric screen. (Nurse practice 2)

They do not want to take the responsibility themselves to say … right I need to address this, this is what I need to do and this is how I'm going to do it. (Nurse practice 12)

There was concern about changes to practice and management that primary care had to incorporate with fears that WISE was yet another initiative that would fizzle out. The training was considered by some to be inappropriate for support staff. Signing up to the training and the link to research meant clinicians expected that they would have to invest additional time with patients generating more work.

They very much operate like businessmen, GPs, in terms of they've got a million and one demands on their time, they've got contracts with the PCT. To hold their contract, they've got to deliver this, and this and this. So when there's something else being thrown in for them to apportion their time and energy to, they're very much going to do a cost-benefit analysis, and if there isn't something very clear, then for some of them, that will just be it. (Manager)

There was negativity towards the inclusion of IBS as some GPs do not code IBS as a diagnosis and reported little difficulty in managing this condition [47]. Additionally, IBS is not a condition within the QOF and thus may not be prioritised by primary care clinicians [48].

#### Acceptability and utility of training

The delivery of training achieved several aims:

1. Engaging a high proportion of practices with the programme, from 51 eligible practices, 44 agreed to participate although three withdrew from training.
2. Delivering training to a high proportion of clinicians and other staff, 90% of eligible staff (*n*?=?179) attended session 1 and 82% (*n*?=?85) attended session 2.
3. Ensuring training was relevant and acceptable, 76% rated session 1 positively and 89% session 2 (see Table 2).

Seemingly, practices were receptive to training as an opportunity for the whole practice to meet, rather than to engage with SMS.

Well I had no objection to it, it was quite nice to have a little sort of team-building day, because we tend to get…I just sit in this little room on my own …… and occasionally see them when they bring me a coffee in, if they remember I'm here. So it was actually quite nice to all sit round and swap a few ideas, when we're all on kind of an equal footing. (GP practice 22)

The trainers reported (in detailed notes and in interviews) on the positive reception to the training and alluded to the problems in putting it into practice.

They all seem to enjoy the training but it's what they do with it is, perhaps, we're not quite clear. (Trainer)

### Embedding SMS in day-to-day routines of primary care: a can of worms

Self-management support was afforded minimal value or priority so little effort was invested by practices in attempting to use the techniques or tools. The guidebooks were reported as being the tools used most regularly in practice, as they fitted with the established role of nurses as educators or 'information-givers' and were minimally disruptive to their consultations.

The PRISMS tool, designed to elicit patients' needs and priorities, was not taken up for regular use—nurses were less likely than GPs to use it. Its use was considered to be too disruptive in terms of QOF tasks (which the practices prioritised) and the maintenance of relationships (which nurses took pride in).

I just think there would be some patients that I'd probably just fear them getting their hands on a PRISMS form, for the amount of work it could create. I know that people have got all these different problems that perhaps we should bring out and try and tackle, but half the time you can't actually tackle those people's problems anyway. And I know that's a fairly negative philosophy on general practice, but there is some truth in it. No, I'm quite fearful of the PRISMS form. (GP, Practice 22)

**Figure 2:**
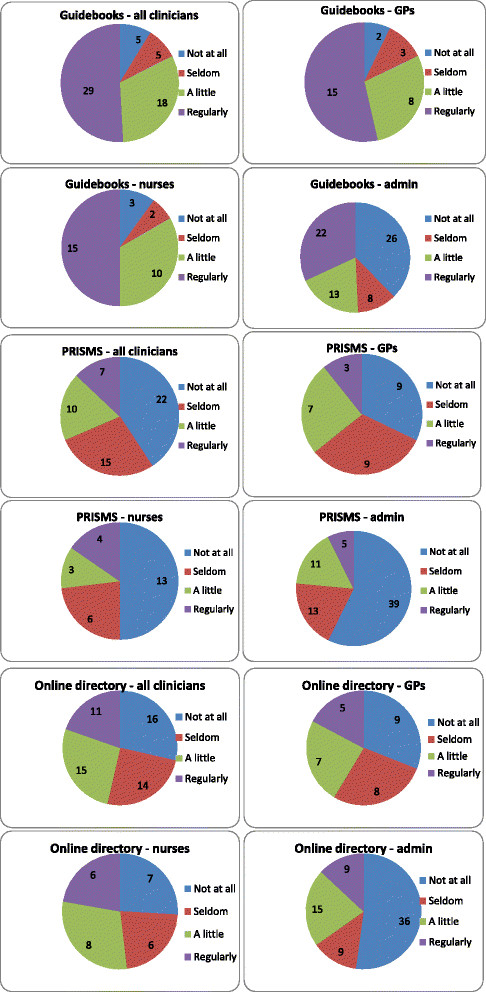
**Use of WISE tools by practice staff.**

The online directory was used most by the nurses (see Figure 2). It had been identified by practices as something they needed to help direct patients to appropriate support yet it was infrequently used as it was hard to navigate and too time-consuming within a consultation.

The RCT findings showed no difference between groups in accessing each type of support indicating that the training had no effect on improving support for patients [24].

Clinicians were not convinced that WISE was different enough to warrant engagement in a new way of working, and the indifference was picked up by the trainers.

I don't know that it brought that much to us really. I think it gave us something, but I don't think it's an awful lot different from what everybody else has been telling us. We need to get people to self-manage everything. (Nurse practice 12)

…I think maybe it's because as a concept it's not defined very well, it's all very vague, so people take from it what they want to take from it. Like a lot of GPs think self-management, you know, they're doing it if they just give out the leaflet, … And self-management is not a priority, and it's something that gets bandied around the PCT a lot but, you know, GPs are like their incentives and direct-payment incentives, the incentives that if we do this it will…(Trainer)

### Patients' uptake and embedding

#### Intervention what intervention?

The interviews explored patients' views on the WISE tools and their experiences and expectations of SMS. Patients were purposefully sampled from practices where staff had reported using PRISMS, and all trial intervention group participants had been sent a PRISMS leaflet in the post. However, none recalled seeing the form prior to the interview so the instruments were introduced to patients during the interviews.

Prior experience indicated that 'forms' would be not worth the effort of completing given the burden of previously experienced forms which seemed to have little personal relevance or meaning.

I would think 'why are they doing that' because they have… I had to fill forms in before at the surgery and nothing has ever come off. Never even been mentioned or, you know? So no, I don't think it would be taken into consideration at all. (Diabetes patient)

No patients considered they would use the form, the only utility being as a memory prompt.

Uppermost in patients' minds was the receptivity of GPs to patient-initiated prompts within a consultation and the feasibility of making this work in practice. Patients were rehearsing the needs of professionals and conscious of the problems of disrupting the status quo of engrained consultation practices.

I haven't used it… if you're like me, you keep forgetting, you can go through it, tick it all off and then just hand it in to your GP when you go… rather than trying to remember what you were going to say when you get there… it's a good idea. But, whether the GPs would like it you don't know, they should do.. (IBS patient)

A good relationship, meant being able to raise concerns easily and being listened to, most likely to occur in nurse consultations. However, for most participants, their relationship ranged from ambivalent to negative, and this influenced the perceived acceptability and workability of PRISMS. Patients, it seems, work out what is on offer in the consultation and the limits of their power to change an expected and engrained response developed over years.

None of the participants reported using the online directory or being referred to it by their GP or practice nurse. Patients did not experience any changes in the nature of engagement with primary care professionals as a result of the intervention.

There was scepticism about the merit in seeking self-management advice from a health care professional as that was viewed as within the province of the patient, and there was minimal experience of the health service encouraging or fostering this [49].

I think to myself, well, if I'm the one that's looking after the condition, I'm the one that's got to put… because what can they do to help me? They can't give me tablets to stop me feeling sluggish when I've been to the toilet. (IBS patient)

At my doctor you go for your reviews, or you do your bloods, it's never how are you getting on… (Diabetes patient)

The majority of participants did not recollect being given a guidebook and reported patchy provision of information from their GP or nurse. Patients who had looked through the guidebooks indicated that they found it helpful to learn how others coped with the condition.

You read them and if there's anything that catches the eye, what I'll do, I do take notice of what people say and what advice…Yeah, they're good. They are useful. (IBS patient)

#### The cycle of a poverty of expectations

There were generally low expectations of support based on previous experiences of difficult and unhelpful relationships and rationed support. People reported avoiding their GPs because of low opinions of the help they would get.

...but when your doctor says something that really doesn't help, you know, and I have had her say "well you just need to control what you put in your bloody mouth" (Diabetes patient)

Review appointments and the focus on monitoring led to expectations that the practice knew about and understood the patient. Reviews were perceived as providing a safety net but there was a lack of shared decision-making.

I just thought hold on a minute there's been no consultation about whether I need to use insulin or not and that just frightened me. (Diabetes patient)

The restrictions imposed by the organisation of primary care militate against meaningful and supportive discussions and add to the lack of confidence in obtaining support:

Apparently you've got ten minutes, but it's like two weeks to get in to see her. So I had a little list, because my memory's not very good with this fibromyalgia. I wanted advice on my IBS, seeing if she could come up with something different while I was in the proceeds of suffering as well as this fibromyalgia, as well as menopause, things like that, but she said right, start with the most important, and then when my ten minutes was up she said would you like to make another appointment please. I thought right, I've got another two weeks to wait. (IBS patient)

In order to obtain appropriate support, patients often had to work hard to persuade the doctor with low expectations of assistance.

I had to go to a pharmacy that does the programme there…you can only purchase it through a pharmacy. And they weigh you every week, they advise you, but they will only let you do the programme with the agreement of the doctor. So they approached my doctor, she was reluctant to do it at first, and it took a month to get her agreement for me to do it. (Diabetes patient)

#### Trust the experts: where self-management support comes from

A lack of trust was evident in the types of support offered by primary care practitioners, mainly because suggestions were not sufficiently thought through or tailored to people's circumstances. The best advice came from personal contacts who had relevant experience [50],[51].

And then [nurse] was on about exercise, so she said, 'Why not go to the gym?' Anyway, she gave me a letter to go to see… [X]… I went to a class and I thought to myself, 'They're all old people here.' Some of them weren't as old as me but the exercises that they were doing I thought, 'That's not enough for me', because before that, the girl next door had said she had gone to this gym. And she said to me, 'It's ladies only…. How about coming with me?' So I started going there with her and I thoroughly enjoyed it (Diabetes patient)

Pharmacists were named as sources of support about medications, particularly in relation to multi-morbidity, but this resulted in tensions. Where many professionals were involved, patients had to work out *whose* advice was most relevant to their circumstances and felt uncomfortable challenging their GP.

She's [pharmacist] just got a very good attitude to the customers and the people that go in and so you can talk to her. But I will talk to her if need be or I will say to her I feel like this, and she knows all the medication I'm on because they deliver it for me, and she'll say, really you need to see your GP because you take this and you take that. Oh can't you just give me something so I don't have to go. (COPD patient)

### Over-arching analysis of components

The integration and enactment of SMS differed according to context. WISE aligned with delivery of policy guidelines to improve the health of the population thus from a top-down organisation perspective; it was easy to buy into (in NPT terms *cognitive participation for initiation*). For practitioners, where WISE could have had the most impact, there was no alignment with practice priorities dominated by the business model of QOF and no perceived relevance or use in providing SMS because of the biomedical focus of chronic disease management (no *legitimation*). Practice nurses performed the prioritised biomedical monitoring tasks related to QOF and could not readily *differentiate* this from SMS. For patients, the context remained the same—there was no change and 'business as usual' concerning their interactions with primary care (no *coherence*). Patients did not view SMS as *legitimate* work to do with their health care professional, so would not initiate discussion or disrupt the status quo.

Expectations of what WISE could deliver were high for the PCT (reduced costs because of a healthier self-managing population (*internalisation*)) but health professionals and patients had low expectations. Health professionals viewed it as a lot of work with no gain for them or their patients (generally viewed as having too many complex needs to be receptive to self-management) and both patients and practitioners found PRISMS potentially disrupting to consultations and well-established practice systems (poor *interactional workability*). Patients did not expect to get support from GPs or nurses in managing day-to-day problems of living with a long-term condition—this came from other sources, family or friends and was tempered by prior experiences of lack of help from primary care. This accords with findings that social deprivation is linked to trust and confidence in GPs [52]. The training was seen as a different approach and difficult to *operationalize* by professionals and middle managers; there was nothing tangible to measure or audit and the individual practice training model viewed as costly and complex, so there were no accounts of *reflexive monitoring*. Trainers were seen as short-term employees and had no management backup. The merits in bringing a practice together were undoubtedly outweighed by the expense and minimal changes to professional behaviours, and the role of admin staff in the training and in SMS was seldom acknowledged or valued. The guidebooks were viewed as the one aspect of the intervention that all found useful because they fitted well with health education work, and adoption was more likely because they improved the perceived patient-centredness of information-giving (good relational integration) [53].

Overall, there was non-alignment over the aspirations most clearly articulated and adopted at the top—but which were not a priority for practitioners and patients despite national policy.

## Discussion

The process evaluation findings help explain why WISE implementation did not lead to SMS becoming embedded in primary care. NPT assisted understanding of what happened across the four levels of WISE: organisational, practice, health professional and patient. Whilst some aspects of implementation worked well (participating in training was a valued opportunity to bring the practice together), the explanations for WISE not embedding at any level were the lack of commitment and views that providing and enacting SMS within primary care practice was not a legitimate activity for patients or clinicians. In addition, during implementation, the PCT was disbanded as part of wider NHS changes so there was a considerable amount of organisational change, loss of staff and shifting priorities. This represented a significant 'outer context' change [23], which could not have been anticipated and had the most impact at the organisational level (where the research team had developed good relationships) but little at the professional or patient levels.

Use of NPT in implementation studies has highlighted similar problems with engaging practitioners in order to embed practice. Lloyd et al. [44] found that work was needed to ensure shared understanding within teams concerning the purpose of shared decision-making with patients. Other process evaluations of RCTs have used NPT to help explain lack of replication of single-centre trials; however, Clarke et al. found that contextual factors including organisational history and team relationships impinged on training implementation, and NPT had limitations in this area because of undue emphasis on individual and collective agency [54]; they also found that an NPT framework does not place 'sufficient emphasis on those who receive complex interventions, especially when the 'service user' is referred to as a 'partner in care.' We would concur with this. There is a need for greater consideration in implementation theory of the importance of the patient role and the implementation work they need to do. Consideration of the Consolidated Framework for implementation [23], in conjunction with NPT, helped in interpreting the wider context—the 'outer setting' in Damschroder's model—and the mismatch between policy and practice.

Why did WISE fail in implementation? The RCT itself was well-implemented, with good reach in terms of practices and patients recruited. How WISE was interpreted and made meaningful in a particular primary care context contributed to a failure to embed the intervention in everyday practice. This came from professional and, to an extent, patient views that self-management support was something that ultimately was not the core business of primary care, and these expectations shaped the fate of the intervention post-training. The challenge is to show how an intervention of greater intensity or duration could enhance effectiveness without compromising reach. The training intervention was well-attended by staff and apparently liked. The complex intervention had good evidence to support its effectiveness. The training was developed with Gask using her evidence-based principles [31],[55]-[57]. However, some of what was done was untested in an RCT, for example, training whole practice teams. Pragmatic changes were needed to roll out the training, namely flexibility, simplification of training and use of lay trainers rather than health professionals—so WISE may have lost potency as an intervention. Unpicking the components of the intervention and considering what was ineffective in the primary care context could enhance understanding. The evidence for training teams in SMS was derived from trials in secondary care. Quality improvement interventions with financial incentives can improve collaboration and patient outcomes in primary care [58],[59]. The training aimed from the outset to instil a learning organisational ethos and to actively engage a range of staff. This was successful as notes from the trainer indicated that in the first WISE sessions, an enjoyable collegial atmosphere was evident which seemingly underpinned the positive scores in the post-training satisfaction survey (Table 3). However, the clear engagement and satisfaction with training did not translate into the practices of everyday working and probably relates to primary care culture and drivers (QOF in the UK). Communication within practices was not changed, and role demarcation and silo working patterns persisted. The lack of didactic direction or direct monitoring may have contributed to lack of uptake together with the lack of identifiable champions in PCT management or in practices to drive the embedding of a culture of SMS [60]. Emerging evidence about implementation has found that audit and feedback are effective in improving quality of care, and such support might be important post-training [61]. The shared decision-making component, PRISMS, was ineffective. Whilst there is evidence that shared decision-making and patient reported outcomes are effective, the obstacle here is the difficulty both clinicians and patients had with integrating PRISMS into consultations.

**Table 3:** **Satisfaction with training**

	***N***	Minimum	Maximum	Mean	Std. deviation
Session 1—all practice staff					
265 participants ranging from 4 to 16 per practice					
??Did you find the training useful	265	1	4	3.05	.820
??Did you like the structure	264	1	4	3.05	.766
??Did you learn from others	264	0	4	3.08	.751
??Was patient pathway useful	263	0	4	2.99	.803
??Was creating opportunities helpful	255	0	4	2.91	.791
??Were the discussions of benefit	263	0	4	3.11	.784
??How actively involved were you	262	1	4	2.96	.772
??Will practice use PRISMS	255	0	4	2.80	1.007
??How likely is system change	252	0	4	2.50	.815
??Valid *N* (listwise)	232				
Session 2—GPs and nurses					
124 participants ranging from 1 to 7 per practice					
??Did you find the training useful	123	1	4	3.21	.668
??Did you like the structure	124	1	4	3.18	.663
??Did you learn from others	124	0	4	3.19	.779
??Was the DVD useful	120	1	4	2.93	.796
??Did you find role play helpful	108	0	4	3.06	.818
??Were the discussions of benefit	124	1	4	3.35	.665
??Will you be able to use the skills	116	1	4	3.26	.674
??Valid *N* (listwise)	100				

Evaluation forms were completed by staff from 31 practices at the end of each training session (18 intervention, 13 control-control practices received training after the 12-month patient follow-up questionnaires had been completed but note that the 6 neighbouring control practices were not trained (outwith the time for the programme grant) and 4 randomised practices withdrew from training).

Score range: 0?=?not at all, 4?=?very much.

## Conclusion

This study contributes to a better understanding of why SMS is not routinely adopted and implemented in primary care, by illuminating how and why providing and enacting SMS was not viewed as a legitimate activity or priority by professionals and organisations. There was a failure to, in principle, engage with and identify patients' support needs. Thus, policy presumptions concerning SMS within primary care appear to be misplaced. Implementation failure brings with it potential to negatively impact on individuals' opportunities to maximise improvements they can make to quality of life in living with a long-term condition. There are also likely significant fiscal costs to the health system if robust SMS is not adopted in primary care given the expectations of decreased utilisation with increased SMS. Implementation of SMS within the health service is bound to fail if it does not account for and connect to patient circumstances and real-life priorities. Finally, self-management requires resources which extend beyond the immediacy of the practices implicating the need for links to broader networks of care and greater understanding of network mechanisms [62]. It is possible to extend systems of resources that involve other agents, but this requires programmatic change.

## Endnotes

^a^PCTs are now defunct, so the term is a historical reference.

^b^One of the senior managers interviewed was the executive responsible for WISE and a member of the steering committee.

## Authors' contributions

AK, AR, CCG, TB and JP contributed to the early conception and theoretical framework for the study design. RB, CG, VL and RM collected data for the process evaluation. AK drafted the manuscript. All authors contributed to the design of instruments and schedules used to collect data and analysis and interpretation of the data, contributed to revising the drafts and gave approval to the final version.

## Additional file

## Electronic supplementary material

Additional file 1: Interview questions.(DOCX 16 KB)

Below are the links to the authors’ original submitted files for images.

Authors’ original file for figure 1

Authors’ original file for figure 2

Authors’ original file for figure 3
