# Supplementary material for: Implementation of a self-management support approach (WISE) across a health system: a process evaluation explaining what did and did not work for organisations, clinicians and patients
Source: Implement Sci. 2014 Oct 21;9:129. doi: 10.1186/s13012-014-0129-5 (PMC4210530; doi:10.1186/s13012-014-0129-5)
Supplement: Supplementary file 1 — Additional file 1: Interview questions.(DOCX 16 KB) [file 13012_2014_129_MOESM1_ESM.docx]

**Additional file**

Interview questions

**Organisations**:

***1. Individual details***

Job title, involvement with WISE.

***2. History of WISE and NIHR***

Knowledge of WISE’s early beginnings.

Knowledge and understanding of NIHR

***3. PCT***

How is WISE being rolled out?

Who’s dealing with it?

How does WISE fit in with other PCT objectives.

Problems – examples

Successes – examples

How do they see interactions with SR?

How does WISE relate to the PCT objective of implementing evidence-based practice more generally?

***4. Wider context***

How do they think WISE relates to the wider context of R&D?

Links to concerns re deprivation and inequalities?

How does WISE mesh with Salford’s identity of a ‘go-ahead’ PCT?

**Health care professionals:**

1. How are patients suffering with COPD, diabetes and IBS managed within your practice?
2. How easy is it to engage patients in self-management techniques?
3. What did you think of the actual training sessions?
4. How did the WISE training differ, if at all, from other training that you have taken part in?
5. Do you think that the training was appropriately targeted at all the staff in the practice?
6. Immediately after the training had been delivered, were the action plans that had been decided upon put into place?
7. By comparison with your existing approach to managing patients with LTCs, did you identify any aspects of the training that you thought could be useful within the practice? Eg PRISMS, guidebooks, online directory
8. To what extent do you think that the training was beneficial to you personally?
9. Has the training resulted in any changes in your consultation technique, or the way that you manage patients who have LTCs?
10. How useful was the training do you think for your practice as a whole?
11. Where changes in practice have been reported …. Have you noticed any changes in patients’ response?
12. In general, do you think that WISE training can offer any benefit in terms of the self care support your practice is able to offer patients?
13. Do you think the WISE training is likely to have any long term impact in this practice?

**Patients:**

1. Current Health and management
2. Relationship with your GP surgery
3. **SHOW / EXPLAIN PRISM FORM** If yes:
   1. How did you come by it?
   2. Has it been used during a consultation with a practitioner?
   3. If so, how was it used?
   4. How effective was it in terms of raising issues and widening the agenda?
   5. Was it beneficial to the consultation process?

If no:

- 1. Do you see this as being something that you personally would find useful as a way of raising issues during a consultation?
  2. Can you think of any circumstances in a recent consultation when this would have been useful?

1. **SHOW INFO BOOKLETS** If yes:
   1. How did you come by it?
   2. Have you read it?
   3. What did you think of it?
2. Your health in the future. Is there anything that you think you could do to help yourself?
