# Supplementary figures and images for: Implementation of a self-management support approach (WISE) across a health system: a process evaluation explaining what did and did not work for organisations, clinicians and patients
Source: Implement Sci. 2014 Oct 21;9:129. doi: 10.1186/s13012-014-0129-5 (PMC4210530; doi:10.1186/s13012-014-0129-5)

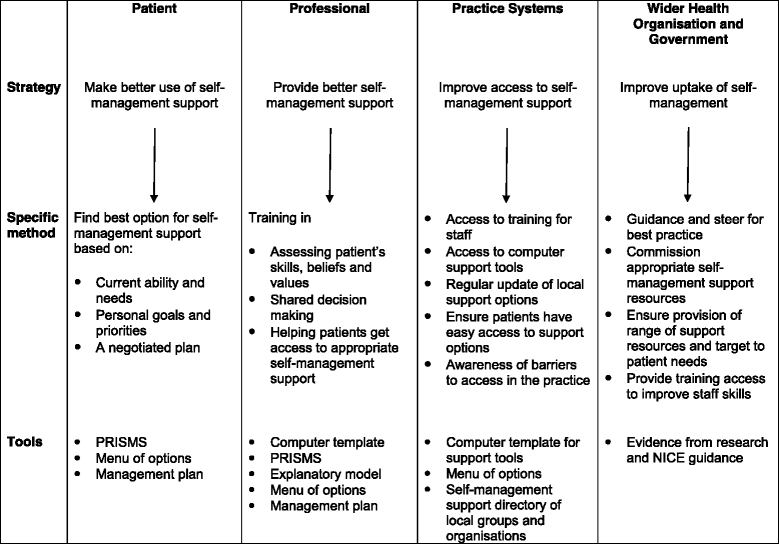

Supplement: Supplementary file 2 — Authors’ original file for figure 1 [file 13012_2014_129_MOESM2_ESM.gif]

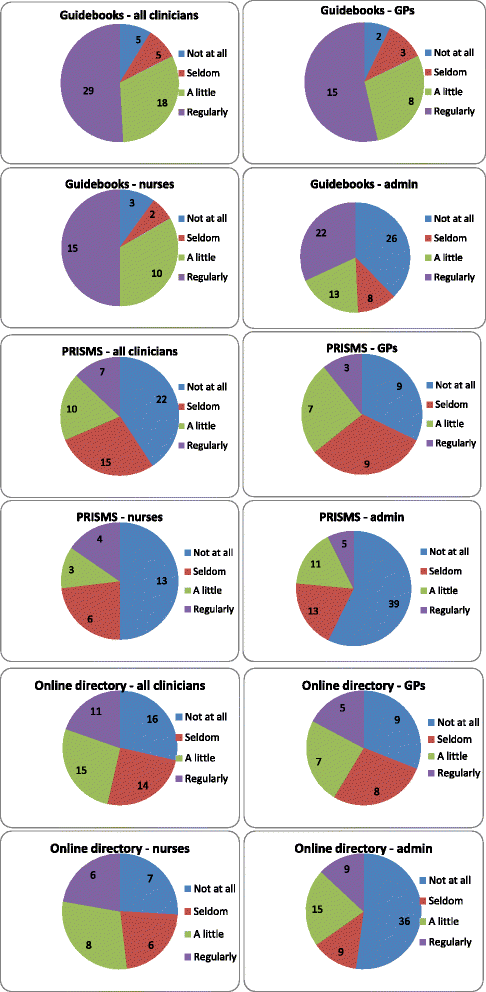

Supplement: Supplementary file 3 — Authors’ original file for figure 2 [file 13012_2014_129_MOESM3_ESM.gif]
