# Supplementary material for: Implementation of a self-management support approach (WISE) across a health system: a process evaluation explaining what did and did not work for organisations, clinicians and patients
Source: Implement Sci. 2014 Oct 21;9:129. doi: 10.1186/s13012-014-0129-5 (PMC4210530; doi:10.1186/s13012-014-0129-5)
Supplement: Supplementary file 4 — Authors’ original file for figure 3 [file 13012_2014_129_MOESM4_ESM.docx]

**Figure 3: Use of WISE tools by practice staff**

The questionnaire survey to determine use of WISE tools was sent to 302 staff at the 31 practices where training took place, 163 administrative staff and 139 clinicians. There was an overall response rate of 48%, 88 admin staff and 67 clinicians.
